# Ipilimumab plus nivolumab and DNA-repair defects in AR-V7-expressing metastatic prostate cancer

**DOI:** 10.18632/oncotarget.25564

**Published:** 2018-06-19

**Authors:** Karim Boudadi, Daniel L. Suzman, Valsamo Anagnostou, Wei Fu, Brandon Luber, Hao Wang, Noushin Niknafs, James R. White, John L. Silberstein, Rana Sullivan, Donna Dowling, Rana Harb, Thomas R. Nirschl, Brendan A. Veeneman, Scott A. Tomlins, Yipeng Wang, Adam Jendrisak, Ryon P. Graf, Ryan Dittamore, Michael A. Carducci, Mario A. Eisenberger, Michael C. Haffner, Alan K. Meeker, James R. Eshleman, Jun Luo, Victor E. Velculescu, Charles G. Drake, Emmanuel S. Antonarakis

**Affiliations:** ^1^ Department of Oncology, Johns Hopkins University School of Medicine, Baltimore, MD, USA; ^2^ Department of Pathology, Johns Hopkins University School of Medicine, Baltimore, MD, USA; ^3^ Department of Urology, Johns Hopkins University School of Medicine, Baltimore, MD, USA; ^4^ Office of Hematology and Oncology Products, Center for Drug Evaluation and Research, US Food and Drug Administration, Silver Spring, MD, USA; ^5^ Department of Pathology, University of Michigan Medical School, Ann Arbor, MI, USA; ^6^ Department of Urology, University of Michigan Medical School, Ann Arbor, MI, USA; ^7^ Epic Sciences Inc., San Diego, CA, USA; ^8^ Department of Hematology/Oncology, Columbia University Medical Center, New York, NY, USA; ^9^ Present address: Pfizer Inc., Pearl River, NY, USA

**Keywords:** AR-V7, DNA repair, ipilimumab, nivolumab, prostate cancer

## Abstract

AR-V7-expressing metastatic prostate cancer is an aggressive phenotype with poor progression-free survival (PFS) and overall survival (OS). Preliminary evidence suggests that AR-V7-positive tumors may be enriched for DNA-repair defects, perhaps rendering them more sensitive to immune-checkpoint blockade. We enrolled 15 metastatic prostate cancer patients with AR-V7-expressing circulating tumor cells into a prospective phase-2 trial. Patients received nivolumab 3 mg/kg plus ipilimumab 1 mg/kg every 3 weeks for four doses, then maintenance nivolumab 3 mg/kg every 2 weeks. Targeted next-generation sequencing was performed to determine DNA-repair deficiency (DRD) status. Outcomes included PSA response rates, objective response rates (ORR), PSA progression-free survival (PSA-PFS), clinical/radiographic PFS and OS. Median age of participants was 65, median PSA was 115 ng/mL, 67% had visceral metastases, and 60% had ≥4 prior systemic therapies. Six of 15 men (40%) had DRD mutations (three in *BRCA2*, two in *ATM*, one in *ERCC4*; none had microsatellite instability). Overall, the PSA response rate was 2/15 (13%), ORR was 2/8 (25%) in those with measurable disease, median PSA-PFS was 3.0 (95%CI 2.1–NR) months, PFS was 3.7 (95%CI 2.8–7.5) months, and OS was 8.2 (95%CI 5.5–10.4) months. Outcomes appeared generally better in DRD+ vs. DRD– tumors with respect to PSA responses (33% vs. 0%; *P*=0.14, nonsignificant), ORR (40% vs. 0%; *P*=0.46, nonsignificant), PSA-PFS (HR 0.19; *P*<0.01, significant), PFS (HR 0.31; *P*=0.01, significant), and OS (HR 0.41; *P*=0.11, nonsignificant). There were no new safety concerns. Ipilimumab plus nivolumab demonstrated encouraging efficacy in AR-V7-positive prostate cancers with DRD mutations, but not in the overall study population.

## INTRODUCTION

Androgen-receptor splice variant 7 (AR-V7) is a constitutively-active isoform of the androgen receptor that is associated with a particularly aggressive form of advanced prostate cancer [1]. Because AR-V7 lacks the androgen-receptor ligand-binding domain, AR-V7-positive prostate cancers are generally resistant to novel hormonal therapies including abiraterone and enzalutamide [2, 3]. In addition, prostate cancers expressing AR-V7 often show poor responses to taxane chemotherapies including docetaxel and cabazitaxel [4, 5]. To this end, patients with AR-V7-positive prostate cancer generally have a median progression-free survival (PFS) of only 3-4 months and a median overall survival (OS) of 7-9 months. Therefore, developing effective therapies for AR-V7-expressing advanced prostate cancer represents an urgent unmet need.

Immune-checkpoint blockade may be one potential strategy to treat such patients. In many cancer types, inhibition of cytotoxic T-lymphocyte–associated antigen 4 (CTLA-4) and/or the programmed death 1 (PD-1) receptor has resulted in meaningful antitumor responses [6]. In some settings, combined blockade of both PD-1 (mediating T-cell exhaustion in peripheral tissues) and CTLA-4 (involved in earlier phases of T-cell activation) has proven more efficacious than inhibition of either pathway alone [7, 8]. Furthermore, tumors harboring DNA mismatch-repair defects or those with hypermutation may be particularly sensitive to immune-checkpoint inhibition [9, 10]. While prostate cancer is generally regarded as a low–mutation-burden tumor [11] and immune-checkpoint blockade has resulted in only modest benefits as a monotherapy [12, 13], recent data have suggested that AR-V7-expressing prostate cancers may be associated with a greater number of DNA-repair gene mutations and a higher mutation load [14].

We hypothesized that metastatic castration-resistant prostate cancer patients with AR-V7-positive circulating tumor cells (CTCs) would be susceptible to treatment with combined immune-checkpoint blockade, and that this approach would be safe and tolerable. We also sought to determine (in an exploratory fashion) whether treatment efficacy was associated with presence of DNA-repair gene mutations. To test these hypotheses, we conducted a phase-2 clinical trial testing ipilimumab plus nivolumab in patients with AR-V7-positive advanced prostate cancer.

## RESULTS

### Patient characteristics

From March 2016 through December 2016, a total of 36 patients underwent clinical-grade AR-V7 testing for eligibility purposes, 26 (72%) had detectable CTCs, and 16 men (44%) were AR-V7-positive. One patient failed screening, leaving 15 patients that comprised our study cohort. Supplementary Table 1 summarizes the baseline characteristics of the study participants. Median age was 65 years, 47% had ECOG performance-status of 1, median PSA was 115 ng/mL, 67% had visceral (liver or lung) metastases, and 60% had received ≥4 prior regimens for metastatic castration-resistant prostate cancer (mCRPC). All patients received at least one dose of the study drugs. At the time of data cutoff (October 2017), median follow-up was 8.6 (range, 1.9–17.9) months, and two patients remained alive.

### Overall clinical outcomes

All patients were evaluable for efficacy (summarized in Table 1, Supplementary Figure 1). Overall, 2 of 15 men (13.3%, 95%CI 3.7–37.9%) achieved a PSA response. Among the 8 patients with measurable soft-tissue disease, the objective response rate (ORR) was 25.0% (95%CI 7.2–59.1%). Median PSA-PFS was 3.0 (95%CI 2.1–NR) months, and median PFS was 3.7 (95%CI 2.8–7.5) months. Three of 15 patients (20.0%, 95%CI 7.1–45.2%) achieved a “durable PFS”. Median OS was 8.2 (95%CI 5.5–10.4) months.

**Table 1:** Overall outcomes for all patients, and according to DNA-repair deficiency (DRD) status

	Overall (N=15)	DRD Negative (N=9)	DRD Positive (N=6)	HR (95%CI)	*P* value
**PSA**_50_**, N (%)** **(95% CI)**	2/15 (13.3%) (3.7–37.9)	0/9 (0%) (0–29.9)	2/6 (33.3%) (9.7–70.0)	–	0.14
**ORR, N (%)** **(95% CI)**	2/8 (25.0%) (7.2–59.1)	0/3 (0%) (0–56.2)	2/5 (40.0%) (11.8–76.9)	–	0.46
**Durable PFS** **(95% CI)**	3/15 (20.0%) (7.1–45.2)	0/9 (0%) (0–29.9)	3/6 (50.0%) (18.8–81.2)	–	0.044
**PSA-PFS (mo),** **(95% CI)**	2.96 (2.07–NR)	2.07 (1.74–NR)	5.82 (4.24–NR)	0.19 (0.06–0.62)	0.0003
**PFS (mo),** **(95% CI)**	3.68 (2.76–7.52)	2.83(1.87–NR)	6.51 (3.88–NR)	0.31 (0.10–0.92)	0.014
**OS (mo),** **(95% CI)**	8.18 (5.52–10.41)	7.23 (3.45–NR)	9.04 (8.18–NR)	0.41 (0.14–1.21)	0.11

NR: upper 95% confidence limit of survival probability not reached.

### DNA-repair defects and outcomes

Six of 15 patients (40%) harbored potentially deleterious somatic and/or germline mutations in a least one DNA-repair gene (Table 2, Supplementary Table 7C), and were considered DNA-repair deficient (DRD+). Patient 3 had a germline *BRCA2* mutation, patient 4 had somatic mutations in both *BRCA2* and *MSH6*, patient 6 had a somatic *ATM* mutation, patient 8 had a germline *BRCA2* and a somatic *FANCM* mutation, patient 9 had a somatic *ATM* mutation, and patient 14 had a somatic *ERCC4* mutation. Baseline characteristics and clinical outcomes of the DRD+ and DRD– patients are summarized in Supplementary Tables 1 and 2. Two patients (3 and 8) had germline mutations in *BRCA2*, and two patients (4 and 8) had biallelic *BRCA2* alterations resulting from LOH of the wild-type allele. No patient demonstrated microsatellite instability. Mean tumor mutational load was estimated at 3.2 (range, 0.8–7.8) mutations/Mb in DRD+ patients and 1.6 (range, 0.8–3.1) mutations/Mb in DRD– patients.

**Table 2:** Summary of DNA-repair deficiency (DRD) status among the 15 patients treated with ipilimumab plus nivolumab

Patient no.	DRD status	DNA-repair gene	Pathogenic DNA-repair mutations	Germline vs. somatic	Loss of heterozygosity (LOH)	MSI markers shifted	Mutational load (muts/Mb)	Source of tumor DNA
1	–	-	-	-	-	N/A	1.1	Plasma
2	–	-	-	-	-	N/A	2.4	Prostate
3	+	*BRCA2*	E1646Qfs^*^23	Germline	No	0/5	1.6	Liver mass
4	+	*BRCA2* *MSH6*	P3189H E192X	Somatic Somatic	Yes No	0/5	7.8	Lymph node
5	–	-	-	-	-	N/A	3.1	Plasma
6	+	*ATM*	D2708N	Somatic	No	0/5	1.6	Lymph node
7	–	-	-	-	-	0/5	1.4	Epidural mass
8	+	*BRCA2* *FANCM*	D3095E R579H	Germline Somatic	Yes No	0/5	0.8	Prostate
9	+	*ATM*	E2039X	Somatic	No	0/5	1.1	Plasma
10	–	-	-	-	-	N/A	1.1	Plasma
11	–	-	-	-	-	0/5	1.3	Prostate
12	–	-	-	-	-	0/5	0.8	Prostate
13	–	-	-	-	-	0/5	1.3	Lymph node
14	+	*ERCC4*	D762V	Somatic	No	0/5	5.6	Lymph node
15	–	-	-	-	-	0/5	1.8	Liver mass

To further examine whether DNA-repair defects are enriched in AR-V7-positive patients, we interrogated the StandUp2Cancer (SU2C) database comprising whole-exome and transcriptome sequencing from 150 mCRPC biopsies [11], of which 143 had adequate RNA yields. Of these, 17.5% of cases (25/143) had AR-V7/AR-FL ratios on RNA sequencing of >10%, and were designated as AR-V7-high; while the remaining 82.5% (118/143) were designated as AR-V7-low. This threshold was set so that the prevalence of an AR-V7-positive tissue-based test would be broadly similar to that of a positive CTC-based AR-V7 test. To this end, pathogenic DRD mutations were found in 36.0% (9/25) of AR-V7-high cases but only in 18.6% (22/118) of AR-V7-low cases (*P*=0.056), suggesting a possible (but non-significant) association between AR-V7 and DNA-repair defects. In the AR-V7-high SU2C cohort, the altered DNA-repair genes were *BRCA2* (x4), *ATM* (x2), *CDK12* (x2) and *MSH2* (x1).

**Figure 1:**
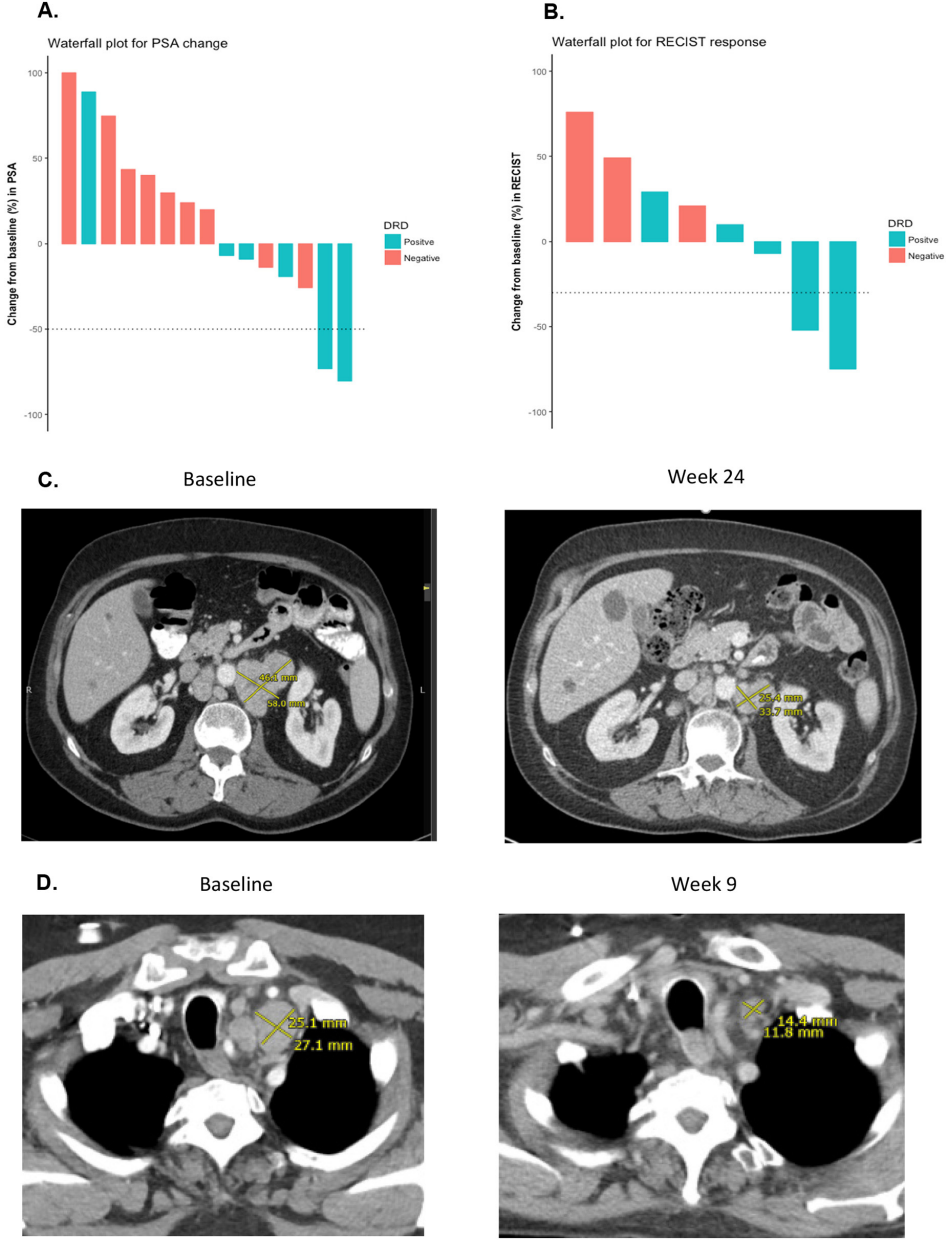
PSA responses and radiographic responses according to DRD status **(A)** Waterfall plot showing PSA responses according to DRD status. The two patients with PSA_50_ responses (#4 and #8) both had biallelic *BRCA2* gene mutations. Patient #4 had a mixed soft-tissue response (some measurable lesions decreased while others increased) and achieved a durable PFS. Patient #8 did not have any measurable disease, but also achieved a durable PFS, and experienced complete resolution of malignant bone pain (pain score 7/10 decreased to 0/10 after 12 weeks of therapy); he is still alive after 17.5+ months of follow-up. **(B)** Waterfall plot showing objective RECIST responses according to DRD status. The two patients with soft-tissue responses (#6 and #14) had mutations in *ATM* and *ERCC4*, respectively. Patient #6 achieved a durable PFS, and is still alive after 17.9+ months of follow-up. **(C)** CT scan of radiographic response for patient #6 (with somatic *ATM* mutation) at baseline and after 24 weeks of treatment. The sum diameter of his target lesions decreased by 52% at the time of his best response. **(D)**. CT scan of radiographic response for patient #14 (with somatic *ERCC4* mutation) at baseline and after 9 weeks of treatment. The sum diameter of his target lesions decreased by 75% at the time of his best response.

**Figure 2:**
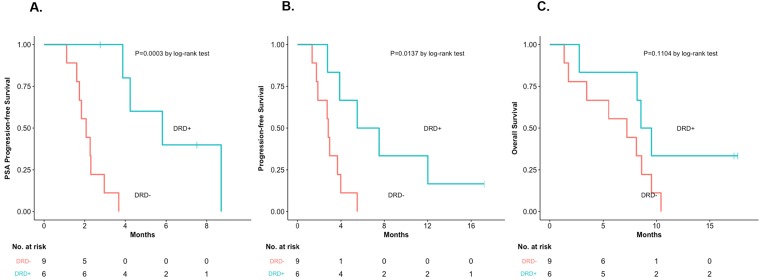
Time-to-event outcomes, according to DRD status **(A)** PSA-PFS, according to DRD status [HR 0.19, 95%CI 0.06–0.62, *P*=0.0003]. **(B)** PFS, according to DRD status [HR 0.31, 95%CI 0.10–0.92, *P*=0.014]. **(C)** OS, according to DRD status [HR 0.41, 95%CI 0.14–1.21, *P*=0.11].

We then compared clinical outcomes in DRD+ and DRD– patients from our trial (Table 1). Response measures appeared generally better in DRD+ versus DRD– cases (Figure 1) with respect to PSA responses (33% vs. 0%; *P*=0.14, nonsignificant), ORR (40% vs. 0%; *P*=0.46, nonsignificant) and “durable PFS” (50% vs. 0%; *P*=0.04, significant). Interestingly, both patients who achieved PSA responses (4 and 8) had biallelic *BRCA2* alterations. Similarly, time-to-event outcomes also appeared better in DRD+ versus DRD– patients (Figure 2) with respect to PSA-PFS (HR 0.19, 95%CI 0.06–0.62; *P*<0.001, significant), PFS (HR 0.31, 95%CI 0.10–0.92; *P*=0.01, significant), and OS (HR 0.41, 95%CI 0.14–1.21; *P*=0.11, nonsignificant).

### Other biomarkers and outcomes

**Figure 3:**
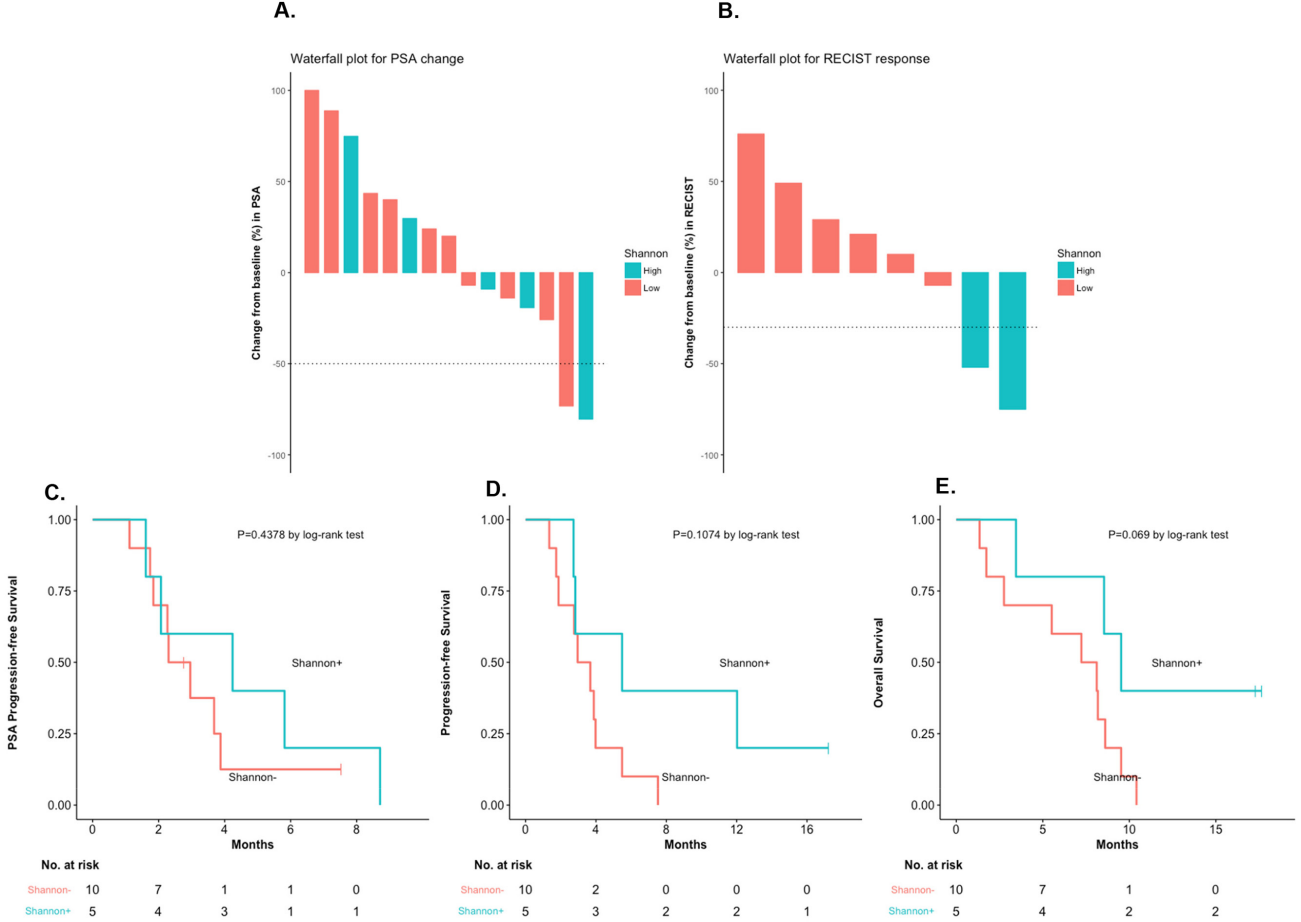
Clinical outcomes, according to Shannon index (low vs. high) **(A)** PSA responses, according to Shannon Index. **(B)** RECIST responses, according to Shannon index. **(C)** PSA-PFS, according to Shannon index [HR 0.67, 95%CI 0.23–1.99, *P*=0.44]. **(D)** PFS, according to Shannon index [HR 0.43, 95%CI 0.15–1.22, *P*=0.11]. **(E)** OS, according to Shannon index [HR 0.34, 95%CI 0.11–0.99, *P*=0.07].

To examine the prognostic impact of CTC phenotypic heterogeneity, we compared outcomes in patients with a high (≥1.5) versus low (<1.5) Shannon index (Supplementary Table 3). Five (33%) and 10 men (67%) were classified as Shannon-high and Shannon-low, respectively. There were numerically more Shannon-high cases among DRD+ compared to DRD– patients (50% [3/6] vs. 22% [2/9] respectively, *P*=0.26, nonsignificant). Outcomes appeared generally better in Shannon-high vs. Shannon-low patients with respect to PSA responses (20% vs. 10%; *P*=1.0, nonsignificant), ORR (100% vs. 0%; *P*=0.04, significant), “durable PFS” (40% vs. 10%; *P*=0.24, nonsignificant), PSA-PFS (HR 0.67, 95%CI 0.23–1.99; *P*=0.44, nonsignificant), PFS (HR 0.43, 95%CI 0.15–1.22; *P*=0.11, nonsignificant), and OS (HR 0.34, 95%CI 0.11–0.99; *P*=0.07, nonsignificant) (Figure 3). Interestingly, both men with RECIST-defined objective responses (6 and 14) had high Shannon indices. CTC pleomorphism (high vs. low) was also assessed in relation to clinical outcomes. No statistical trends were observed (Supplementary Table 4, Supplementary Figure 2), although both patients with PSA responses (4 and 8) were classified as pleomorphism-high.

Eight patients underwent new metastatic biopsies and were evaluable for PD-L1 status. Five (62%) and 3 men (38%) were PD-L1–positive and -negative, respectively. Representative immunostains are shown in Supplementary Figure 3. There were numerically more PD-L1–positive cases among DRD+ compared to DRD– tumors (80% [4/5] vs. 33% [1/3] respectively, *P*=0.19). No statistical trends between PD-L1 status and clinical outcomes were observed (Supplementary Table 5, Supplementary Figure 4), although both patients with objective responses (6 and 14) had PD-L1–expressing tumors.

### Safety and adverse events

The most common toxicities that developed during or after treatment were fatigue, AST elevation, diarrhea and anorexia (Supplementary Table 6). Seventeen grade 3-4 adverse events occurred in 7 of 15 patients (46%). There were two cases of grade 3-4 fatigue, two cases of grade 3-4 diarrhea/colitis, and two cases of grade 3-4 elevated lipase. Immune-related adverse events were of particular interest. There were five events (affecting 33% of patients) that were possibly or probably related to autoimmune phenomena and that required treatment with corticosteroids: two episodes of colitis, two episodes of pneumonitis, and one episode of hepatitis; hypophysitis was not observed. There were no treatment-related deaths.

## DISCUSSION

Prostate cancer expressing AR-V7 represents a lethal phenotype with inadequate treatment options. Here, we report data from the first trial specifically targeting AR-V7-positive disease and the first trial of ipilimumab plus nivolumab in prostate cancer. Although sufficient clinical activity was not observed in the overall study population (and the primary endpoint was not met), encouraging clinical activity using combined immune-checkpoint blockade was seen in the subset of patients harboring germline and/or somatic mutations in DNA-repair genes (and not restricted to mismatch-repair genes). Moreover, there appeared to be a positive correlation between AR-V7 detection and the presence of sequence alterations in DNA-repair genes, further supporting an immunotherapy approach in these patients.

It is now appreciated that approximately 20-25% of metastatic prostate cancers harbor somatic mutations involving DNA-repair genes, primarily homologous-recombination repair genes (e.g. *BRCA2*, *ATM*) and, to a lesser extent, mismatch-repair genes (e.g. *MSH2*, *MSH6*) [11, 21]. The current study, coupled with our secondary analysis of the StandUp2Cancer dataset, suggests that DNA-repair defects (DRD) may be further enriched in AR-V7-positive prostate cancers with a prevalence approaching 40%. These DRD+ patients may benefit from alternative treatment strategies including poly–ADP-ribose polymerase (PARP) inhibitors [22] or other genetically-targeted approaches [23, 24]. The potential association between AR-V7 detection and DRD mutations has also been suggested by a previous study,[14] but still requires further confirmation.

The correlation between DNA mismatch-repair deficiency (resulting in microsatellite instability) and responsiveness to PD-1 inhibitor therapy is now well established, although MMR mutations are only observed in 2-3% of advanced prostate cancers [9]. Our data suggest that sensitivity to immune-checkpoint inhibitors may perhaps be expanded to other types of DNA-repair alterations, particularly homologous-recombination deficiency (HRD) mutations. Among the six DRD+ patients in this study, five had HRD lesions (three in *BRCA2*, two in *ATM*) and one had a nucleotide-excision repair (*ERCC4*) lesion. Interestingly, mean tumor mutational burden was approximately 2-fold higher in DRD+ versus DRD– cancers, although none of these patients demonstrated microsatellite instability. These findings are consistent with two prior studies (including one in prostate cancer) that reported a modestly higher mutational load in *BRCA2*-mutant vs. wild-type tumors [25, 26]. Two other studies in *BRCA1*-deficient breast cancers and *BRCA1/2*-deficient ovarian cancers, respectively, demonstrated that these tumors may have higher predicted neoantigen loads, more tumor-infiltrating lymphocytes and increased expression of PD-1 and CTLA-4 as compared to their homologous-repair–proficient counterparts [27, 28]. Furthermore, a recent study combining durvalumab (a PD-L1 inhibitor) with olaparib (a PARP inhibitor) in mCRPC patients reported high response rates in men with HRD mutations [29]. Finally, a recent clinical study in advanced urothelial carcinoma suggested that outcomes to PD-1 or PD-L1 inhibitors were superior in patients with vs. without HRD mutations [30]. Taken together, these data imply that HRD alterations, not just MMR alterations, may sensitize patients to immune-checkpoint blockade. In addition, the current study is the first to suggest that defects in nucleotide-excision repair (e.g. *ERCC4*) may also be associated with immunotherapy sensitivity.

We also observed a trend between high phenotypic CTC heterogeneity (Shannon index) and favorable responses to combination immunotherapy. In addition, DRD+ patients demonstrated a trend towards higher CTC heterogeneity compared to DRD– patients. Previous studies showed that mCRPC patients with Shannon-high CTCs respond poorly to novel hormonal therapies and better to taxane chemotherapies [20]. Interestingly, the two patients with the highest Shannon indices (6 and 14) both had objective tumor responses, both harbored DRD alterations, and both expressed PD-L1. This suggests a theoretical model whereby DRD mutations result in greater genomic heterogeneity, manifesting as greater phenotypic CTC heterogeneity, and increasing the likelihood of a favorable response to immune-checkpoint inhibition. This hypothesis remains to be proven.

In conclusion, our data suggest that the combination of nivolumab plus ipilimumab demonstrates acceptable safety and encouraging efficacy in men with AR-V7-expressing advanced prostate cancer who also harbor DNA-repair alterations, but not in the overall study population. Moreover, the prevalence of these DNA-repair defects appears to be higher in AR-V7-positive patients. Both of these findings require large-scale prospective validation.

## MATERIALS AND METHODS

### Patient eligibility

Eligible patients had histologically confirmed, progressive, metastatic castration-resistant prostate cancer (mCRPC) with detectable AR-V7 transcripts using the Johns Hopkins CTC-based clinical-grade AR-V7 assay (see below) [15, 16]. Additional eligibility criteria included an ECOG performance-status of 0-1, at least 18 years of age, serum testosterone <50 ng/dL with ongoing androgen-deprivation therapy, adequate organ (liver, kidney, bone marrow) function, and availability of new or archival tumor tissue for biomarker analysis. Key exclusion criteria included a second active malignancy within 5 years, prior immune-checkpoint inhibitor therapy, active brain or meningeal metastases, history of autoimmune disease, or requirement for systemic corticosteroids. Complete eligibility criteria are available in the Supplementary Materials.

### Study design

This was a single-institution one-arm open-label phase 2 study conducted at Johns Hopkins. Patients received treatment by intravenous infusion consisting of 3 mg per kilogram of nivolumab plus 1 mg per kilogram of ipilimumab every 3 weeks for 4 doses, followed by a maintenance regimen of 3 mg per kilogram of nivolumab every 2 weeks thereafter. Treatment continued until radiographic progression, unequivocal clinical progression, development of unacceptable toxicity, or withdrawal of consent. Suspected immune-related toxicities were managed using available guidelines. Patients were not permitted to receive nivolumab maintenance therapy unless they tolerated all four doses of combination immunotherapy.

The primary endpoint was the PSA response rate, defined as a ≥50% decline in PSA from baseline maintained for ≥4 weeks. Secondary endpoints included freedom-from-PSA-progression (PSA-progression-free-survival; PSA-PFS), freedom-from-clinical/radiographic-progression (progression-free-survival; PFS), objective response rate (ORR) according to RECIST1.1 criteria [17] in patients with measurable disease, PFS lasting >24 weeks (termed “durable PFS”), and overall survival (OS). PSA-progression was defined as a ≥25% increase in PSA from baseline or nadir, requiring confirmation ≥4 weeks later (PCWG2 criteria [18]). Clinical/radiologic-progression was defined as unequivocal symptomatic progression (worsening disease-related symptoms or new cancer-related complications), or radiographic progression (CT scan showing ≥20% enlargement in sum diameter of soft-tissue target lesions [RECIST1.1]; bone scan showing ≥2 new osseous lesions not related to bone flare) or death, whichever occurred first. Safety and adverse effects were also assessed.

Study assessments were prospectively defined. PSA measurements were obtained at baseline and every 4 weeks on study. Radiographic evaluations (CT of chest/abdomen/pelvis and technetium-99 bone scans) were performed at baseline and every 12 weeks. Physical examination, toxicity assessments, and laboratory studies (complete blood count, comprehensive metabolic panel, thyroid function) were performed every 4 weeks. Safety was assessed by collecting and grading adverse events according to CTCAE v4.0 criteria.

This was an investigator-initiated trial (NCT02601014) designed by the principal investigators (E.S.A. and C.G.D.) and funded by Bristol Myers-Squibb who also provided both study drugs free of cost. The study was approved by the Johns Hopkins University IRB, and was overseen by an independent scientific review committee and an independent data and safety monitoring committee. All patients provided written informed consent before participation.

### DNA sequencing

All 15 patients underwent prospective tumor DNA sequencing. Details of targeted next-generation sequencing methods performed on pre-treatment tumor, matched normal and circulating-tumor (ct)DNA samples, and bioinformatic analyses, are provided in the Supplementary Materials. We performed targeted sequencing on 8 matched tumor-normal and 3 tumor-only cases (Supplementary Table 7A). In 4 patients, where tumor tissue was not available, we performed next-generation sequencing of cell-free ctDNA (Supplementary Table 7B). In addition to examining sequence alterations and microsatellite instability, we generated estimates of mutation burden for each tumor. We subsequently focused on sequence alterations in DNA-repair genes, identified somatic and germline variants and assessed allele-specific copy-number and loss-of-heterozygosity events for these loci. Putative pathogenic variants were determined by an ensemble of bioinformatic platforms, as described in the Supplementary Materials. To correlate genomic findings with clinical outcomes, patients were classified as “positive” or “negative” for potentially pathogenic mutations in DNA-repair genes. Patients were considered to be DNA repair-deficient (DRD-positive [DRD+]) if they had at least one pathogenic mutation in a gene involved in DNA-damage repair [22]; otherwise they were classified as DRD-negative (DRD–).

### AR-V7 and CTC analyses

A modified AdnaTest assay (Qiagen, Hannover, Germany) conducted in our CLIA-certified laboratory was used to interrogate CTCs for AR-V7 mRNA detection [15], and a positive test was required for eligibility. Briefly, this employs EpCAM-based CTC capture followed by multiplexed reverse-transcription polymerase-chain-reaction (qRT-PCR) using custom primers to detect full-length androgen receptor (AR-FL) mRNA and AR-V7 mRNA, as previously described [2, 15]. In addition, all patients underwent collection of CTCs at baseline using the Epic Sciences platform (San Diego, CA) [19], and these cells were analyzed for phenotypic heterogeneity (Shannon index)[20] and degree of pleomorphism, as described in the Supplementary Materials. Clinical outcomes were compared among patients with high versus low CTC heterogeneity and high versus low pleomorphism.

### PD-L1 analysis

In patients undergoing a new metastatic tumor biopsy, expression of PD-L1 protein was assessed using immunohistochemistry (rabbit monoclonal antibody, Ventana, Tucson, AZ), as described in the Supplementary Materials. A positive test was defined as any percentage of PD-L1 staining on tumor cells.

### Statistical analyses

The primary endpoint was PSA response, and a response rate above 5% was considered clinically meaningful in this AR-V7-positive population. Accordingly, a sample size of 15 patients with ≥3 PSA responses would produce a 90% confidence interval of 6–44%, which would be above the 5% threshold. A positive study would therefore be defined as ≥3 of 15 patients achieving a PSA response.

Analyses of response endpoints (*e.g.* PSA response, ORR) were expressed as proportions with 2-sided Wilson binomial 95% confidence intervals. Time-to-event endpoints (*e.g.* PFS, OS) were analyzed using the Kaplan-Meier method and 95% confidence intervals were generated using the generalized Brookmeyer-Crowley method after log-transformation. Clinical outcomes were compared among patients who were DRD+ and DRD– (primary biomarker analysis), as well as according to other biomarker categories (CTC heterogeneity, CTC pleomorphism, tumor PD-L1 expression). To examine associations between clinical outcomes and biomarker status, response endpoints were compared using Fisher’s exact test, and time-to-event endpoints were compared using the log-rank test with Cox proportional-hazards models to derive hazard ratios. All tests were two-sided, and *P* values ≤0.05 were considered significant; we did not correct for multiple hypotheses. Statistical analyses were performed using *R* (version 3.4.3).

## SUPPLEMENTARY MATERIALS FIGURES AND TABLES




