# Supplementary material for: Ipilimumab plus nivolumab and DNA-repair defects in AR-V7-expressing metastatic prostate cancer
Source: Oncotarget. 2018 Jun 19;9(47):28561–71. doi: 10.18632/oncotarget.25564 (PMC6033362; doi:10.18632/oncotarget.25564)
Supplement: Supplementary file 1 [file oncotarget-09-28561-s001.pdf]

Ipilimumab plus nivolumab and DNA-repair defects in AR-V7-expressing metastatic prostate cancer

SUPPLEMENTARY MATERIALS

METHODS

Targeted next-generation sequencing of tumor samples, and data analyses

Sample preparation, library construction, targeted capture, next-generation sequencing (NGS) and bioinformatic analyses of tumor and normal samples (Supplementary Table 7A) were performed as previously described [1]. Briefly, DNA was extracted from matched FFPE and normal tissue samples using the Qiagen DNA FFPE Tissue Kit and Qiagen DNA Blood Mini Kit respectively (Qiagen GmbH). For three patients, normal tissue was not available and a tumor-only analysis was performed. Genomic DNA from tumor and normal samples was fragmented and used for Illumina TruSeq library construction. Targeted regions of interest were captured usingAgilent SureSelect in-solution capture reagents and a custom-targeted panel for genes of interest (CancerSELECT™ 203, CancerSELECT™ 125, CancerSELECT-R™ 88, Personal Genome Diagnostics [PGDx], Baltimore, MD). Paired-end sequencing, resulting in 100 bases from each end of the fragment for targeted libraries, was performed using Illumina HiSeq (Illumina). Primary processing of NGS data and identification of putative somatic mutations was completed using Illumina CASAVA software (version 1.8) and VariantDx custom software, respectively, as previously described [1]. For patient #2, targeted NGS was performed using the FoundationOne® panel (Foundation Medicine, Boston, MA).

Next-generation sequencing of cell-free DNA, and data analyses

ctDNA was extracted from plasma samples for four patients with no available tumor tissue (Supplementary Table 7B). DNA was isolated from plasma using the Qiagen Circulating Nucleic Acids Kit (Qiagen GmbH). Genomic libraries and targeted capture was performed as previously described [2] for a custom targeted panel of genes (PlasmaSELECT™ 64, Personal Genome Diagnostics [PGDx], Baltimore, MD). Libraries were sequenced using 100-bp paired-end runs on the Illumina HiSeq 2000/2500 (Illumina). Primary processing of NGS data for cfDNA samples was performed using Illumina CASAVA (Consensus Assessment of Sequence and Variation) software (version 1.8). Sequence reads were aligned against the human reference genome (version hg19) using NovoAlign with additional realignment of select regions using the Needleman-Wunsch method.[1] Next, candidate somatic mutations, consisting of point mutations, small insertions and deletions, were identified using VariantDx [1] across the targeted regions of interest.

Microsatellite instability (MSI) status

Microsatellite instability status was determined from targeted NGS using five well-characterized mononucleotide sequences (BAT-25, BAT-26, NR-21, NR-24 and MONO-27, Personal Genome Diagnostics [PGDx], Baltimore, MD).

Tumor mutation burden estimates

Genomic regions of interest associated with somatic mutation identification from 4 targeted gene panels (PlasmaSELECT™ 64, CancerSELECT-R™ 88, CancerSELECT™ 125, CancerSELECT™ 203) were subjected to an *in silico* study of total exome mutational burden and panel-specific mutational load. TCGA exome-based somatic mutations identified by the Broad GDAC Firehose (gdac.broadinstitute.org) from 7,180 patient tumors were collected, requiring a minimum of 4 supporting reads and a 10% mutant allele frequency. Mutations present within each panel's region of interest were aggregated to compute *in silico* panel loads for each patient. For each panel, we then computed moving median values of total mutational load across a sliding window of log-transformed panel loads (each window position included values within ± 0.2 log10 panel load). These moving median values were used as an estimate for expected total mutational burden per exome given a specific panel load. To derive the estimated number of mutations/Mb, the total mutational burden per exome was divided by 50.

Loss-of-heterozygosity (LOH) evaluation

Germline SNPs were identified in the matched normal sample of each patient using GATK HaplotypeCaller (version 3.7) [3] by setting the minimum

phred-scaled confidence threshold to 30. The candidate set of SNPs were narrowed down to those with minimum coverage of 10x, allele count of 1 (heterozygous), quality score normalized by allelic depth (QD) above 2, Fisher's strand bias (FS) below 60, mapping quality (MQ) greater than 40, mapping quality rank sum (MQRankSum) greater than 12.5, and read position rank sum (ReadPosRankSum) greater than 8.0. Minor allele frequency of heterozygous SNPs was calculated in each tumor sample using samtools mpileup module [4]. Genomic regions at constant minor allele frequency (and presumably copy number) were established by application of circular binary segmentation [5].

Biallelic alteration of DNA-repair genes harboring potentially deleterious germline or somatic mutations was evaluated by comparing the minor allele frequency of germline heterozygous SNPs in tumor and normal samples. Segmental minor allele frequency values of 0.35 or below in tumor samples (equivalent to a tumor/normal difference of 0.1) indicated allelic imbalance and therefore loss-of-heterozygosity (LOH). Furthermore, allele-specific copy number was determined to rule out potential cases with allelic imbalance, but no LOH. Briefly, coverage density of reads mapping to target and off-target regions in the genome were compared between tumor and normal samples using CNVKit [6], and corrected for normal contamination as previously described [7]. This process yielded estimates of total integer copy number across the genome. For genomic segments overlapping mutations in DNA-repair genes, all combinations of allele specific copy numbers were examined and the one with the best match to germline SNP allele frequencies was selected. We applied this approach to 5 out of 6 cases with potentially pathogenic mutations in DNA-repair genes (Table 2). For patient #9, the smaller target region of the gene panel precluded detection of a sufficient number of SNPs, therefore LOH could not be evaluated.

Prediction of mutation pathogenicity for DNA-repair genes

All mutations detected in DNA-repair genes, regardless of germline or somatic status, were analyzed with respect to their impact on gene function. Nonsense single-base substitutions and frameshift indels were assumed to disrupt gene function, and were labeled as pathogenic. The impact of missense single-base substitutions, which accounted for many of the DNA-repair gene mutations detected in this study, was bioinformatically predicted by REVEL [8]. REVEL is an ensemble method which evaluates pathogenicity of rare missense variants, based on assessments made by a large number of mutation impact prediction tools developed in the bioinformatics community over the past decade. REVEL scores were retrieved using Ensembl Variant Effect Predictor [9]. Mutations with a REVEL score of 0.5 or above were labeled as pathogenic; this threshold ensures a sensitivity of 0.75 and a specificity of 0.89 for predicting pathogenicity.

CTC detection (Epic Sciences)

Circulating tumor cell (CTC) identification was performed as described previously [10–12]. Briefly, blood from each subject was collected in Streck tubes, shipped to Epic Sciences (San Diego, CA) and processed within 48 hours in a laboratory certified under the Clinical Laboratory Improvement Amendment (CLIA) act. After red blood cell lysis, approximately 3×10^6 nucleated cells were dispensed on up to 12 glass microscope slides and placed at -80°C for long-term storage. Upon testing, slides were thawed and underwent automated immunofluorescent staining for DAPI (nuclear DNA) cytokeratins (CK), CD45, and the N-terminal region of the androgen receptor protein (AR N-term). Every nucleated object on the slides was imaged, and CTCs were identified by a combination of morphological features and malignant biomarkers (CK and AR N-term) in the absence of CD45 expression. Identified CTCs were then subjected to additional digital pathology assessments, requisite for CTC phenotypic heterogeneity assessments. Two slides were tested per sample (corresponding to~1mL of blood) with final quality control conducted by clinical laboratory scientists licensed in the state of California.

CTC phenotypic heterogeneity (Shannon index) and pleomorphism index

CTC phenotypic heterogeneity was assessed using both the Shannon index and Pleomorphism index, as previously described with the Epic Sciences platform [12]. The Shannon index makes use of unsupervised clustering boundaries [12] to define discrete phenotypic subtypes needed for application of a Shannon index. From this original training set, a k-Nearest Neighbor classifier was trained to predict the k-means cluster assignment for every new CTC encountered in the current cohort. Every CTC detected in our cohort was categorized as one of these subtypes, and the collection of these subtypes per patient sample tested was utilized as part of a Shannon index. Consistent with our previous publication [12], a high Shannon index was one with a score of ≥1.5 and a low Shannon index was one with a score of <1.5. The Pleomorphism index [12] utilizes a combined measure of the coefficients of variation of four protein and phenotypic features across CTCs from within a patient sample, and does not make use of discrete phenotypic subtypes. Consistent with our previous publication [12], a high Pleomorphism index was one with a score of ≥2.5 and a low Pleomorphism index was one with a score of <2.5.Therefore, Shannon index is a measure of phenotypic entropy, and Pleomorphism index is a measure of phenotypic variance.

Immunohistochemistry for PD-L1

PD-L1 immunostaining was carried out on an automated instrument (BenchMark Ultra, Ventana medical systems, Tucson, AZ). Slides were deparaffinized, hydrated and heat-induced antigen retrieval was performed with a high pH buffer (CC1, 56 minutes, Ventana). The PD-L1 antibody (rabbit monoclonal clone SP263, REF# 790-4905, Ventana) was applied for 16 minutes at room temperature and immunoreactivity was visualized using the Opti-View detection system (Ventana). Tonsil tissue as well as cancer cell lines with known PD-L1 expression levels were used as positive controls. For each biopsy, at least 100 cancer cells were evaluated. Any amount of cancer cell-specific PD-L1 immunoreactivity was considered a positive test.

REFERENCES

1. Jones S, Anagnostou V, Lytle K, Parpart-Li S, Nesselbush M, Riley DR, Shukla M, Chesnick B, Kadan M, Papp E, Galens KG, Murphy D, Zhang T, et al. Personalized genomic analyses for cancer mutation discovery and interpretation. Sci Transl Med. 2015; 7:283ra53. https://doi.org/10.1126/scitranslmed.aaa7161.
2. Phallen J, Sausen M, Adleff V, Leal A, Hruban C, White J, Anagnostou V, Fiksel J, Cristiano S, Papp E, Speir S, Reinert T, Orntoft MW, et al. Direct detection of early-stage cancers using circulating tumor DNA. Sci Transl Med. 2017; 9:aan2415. https://doi.org/10.1126/scitranslmed.aan2415.
3. Van der Auwera GA, Carneiro MO, Hartl C, Poplin R, Del Angel G, Levy-Moonshine A, Jordan T, Shakir K, Roazen D, Thibault J, Banks E, Garimella KV, Altshuler D, et al. From FastQ data to high confidence variant calls: the Genome Analysis Toolkit best practices pipeline. Curr Protoc Bioinformatics. 2013; 43:1–33.
4. Li H, Handsaker B, Wysoker A, Fennell T, Ruan J, Homer N, Marth G, Abecasis G, Durbin R, and 1000 Genome Project Data Processing Subgroup. The Sequence Alignment/Map format and SAMtools. Bioinformatics. 2009; 25:2078–79. https://doi.org/10.1093/bioinformatics/btp352.
5. Olshen AB, Venkatraman ES, Lucito R, Wigler M. Circular binary segmentation for the analysis of array-based DNA copy number data. Biostatistics. 2004; 5:557–72. https://doi.org/10.1093/biostatistics/kxh008.
6. Talevich E, Shain AH, Botton T, Bastian BC. CNVkit: Genome-Wide Copy Number Detection and Visualization from Targeted DNA Sequencing. PLOS Comput Biol. 2016; 12:e1004873. https://doi.org/10.1371/journal.pcbi.1004873.
7. Anagnostou V, Smith KN, Forde PM, Niknafs N, Bhattacharya R, White J, Zhang T, Adleff V, Phallen J, Wali N, Hruban C, Guthrie VB, Rodgers K, et al. Evolution of Neoantigen Landscape during Immune Checkpoint Blockade in Non-Small Cell Lung Cancer. Cancer Discov. 2017; 7:264–76. https://doi.org/10.1158/2159-8290.CD-16-0828.
8. Ioannidis NM, Rothstein JH, Pejaver V, Middha S, McDonnell SK, Baheti S, Musolf A, Li Q, Holzinger E, Karyadi D, Cannon-Albright LA, Teerlink CC, Stanford JL, et al. REVEL: An Ensemble Method for Predicting the Pathogenicity of Rare Missense Variants. Am J Hum Genet. 2016; 99:877–85. https://doi.org/10.1016/j.ajhg.2016.08.016.
9. McLaren W, Gil L, Hunt SE, Riat HS, Ritchie GR, Thormann A, Flicek P, Cunningham F. The Ensembl Variant Effect Predictor. Genome Biol. 2016; 17:122. https://doi.org/10.1186/s13059-016-0974-4.
10. Beltran H, Jendrisak A, Landers M, Mosquera JM, Kossai M, Louw J, Krupa R, Graf RP, Schreiber NA, Nanus DM, Tagawa ST, Marrinucci D, Dittamore R, Scher HI. The initial detection and partial characterization of circulating tumor cells in neuroendocrine prostate cancer. Clin Cancer Res. 2016; 22:1510–19. https://doi.org/10.1158/1078-0432.CCR-15-0137.
11. Scher HI, Lu D, Schreiber NA, Louw J, Graf RP, Vargas HA, Johnson A, Jendrisak A, Bambury R, Danila D, McLaughlin B, Wahl J, Greene SB, et al. Association of AR-V7 on circulating tumor cells as a treatment-specific biomarker with outcomes and survival in castration-resistant prostate cancer. JAMA Oncol. 2016; 2:1441–49. https://doi.org/10.1001/jamaoncol.2016.1828.
12. Scher HI, Graf RP, Schreiber NA, McLaughlin B, Jendrisak A, Wang Y, Lee J, Greene S, Krupa R, Lu D, Bamford P, Louw JE, Dugan L, et al. Phenotypic heterogeneity of circulating tumor cells informs clinical decisions between AR signaling inhibitors and taxanes in metastatic prostate cancer. Cancer Res. 2017; 77:5687–98. https://doi.org/10.1158/0008-5472.CAN-17-1353.

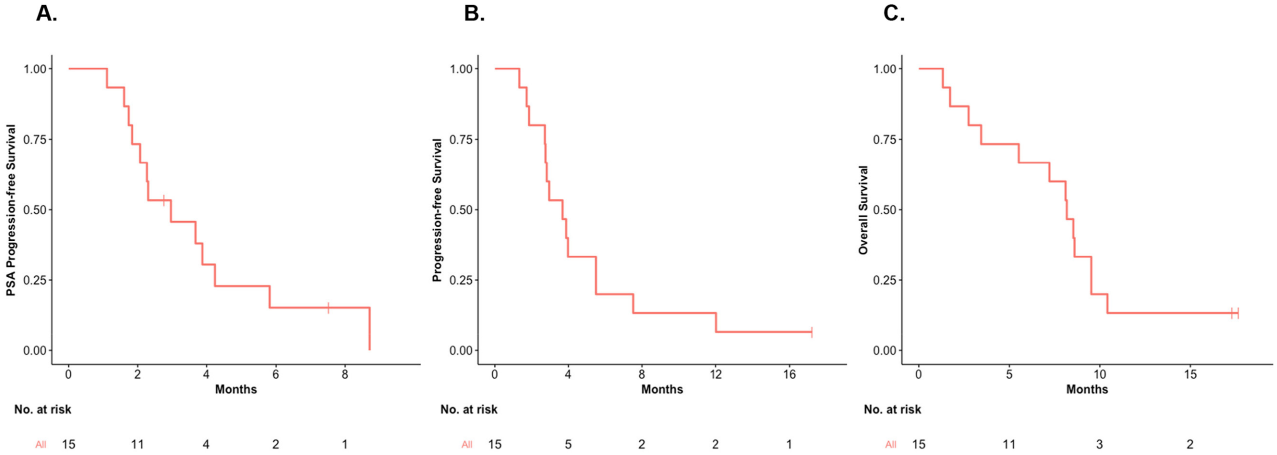

Supplementary Figure 1: Time-to-event outcomes for the overall population. **(A)** PSA-PFS, overall [median 3.0 months, 95%CI 2.1–NR months]. **(B)** PFS, overall [median 3.7 months, 95%CI 2.8–7.5 months]. **(C)** OS, overall [median 8.2 months, 95%CI 5.5–10.4 months].

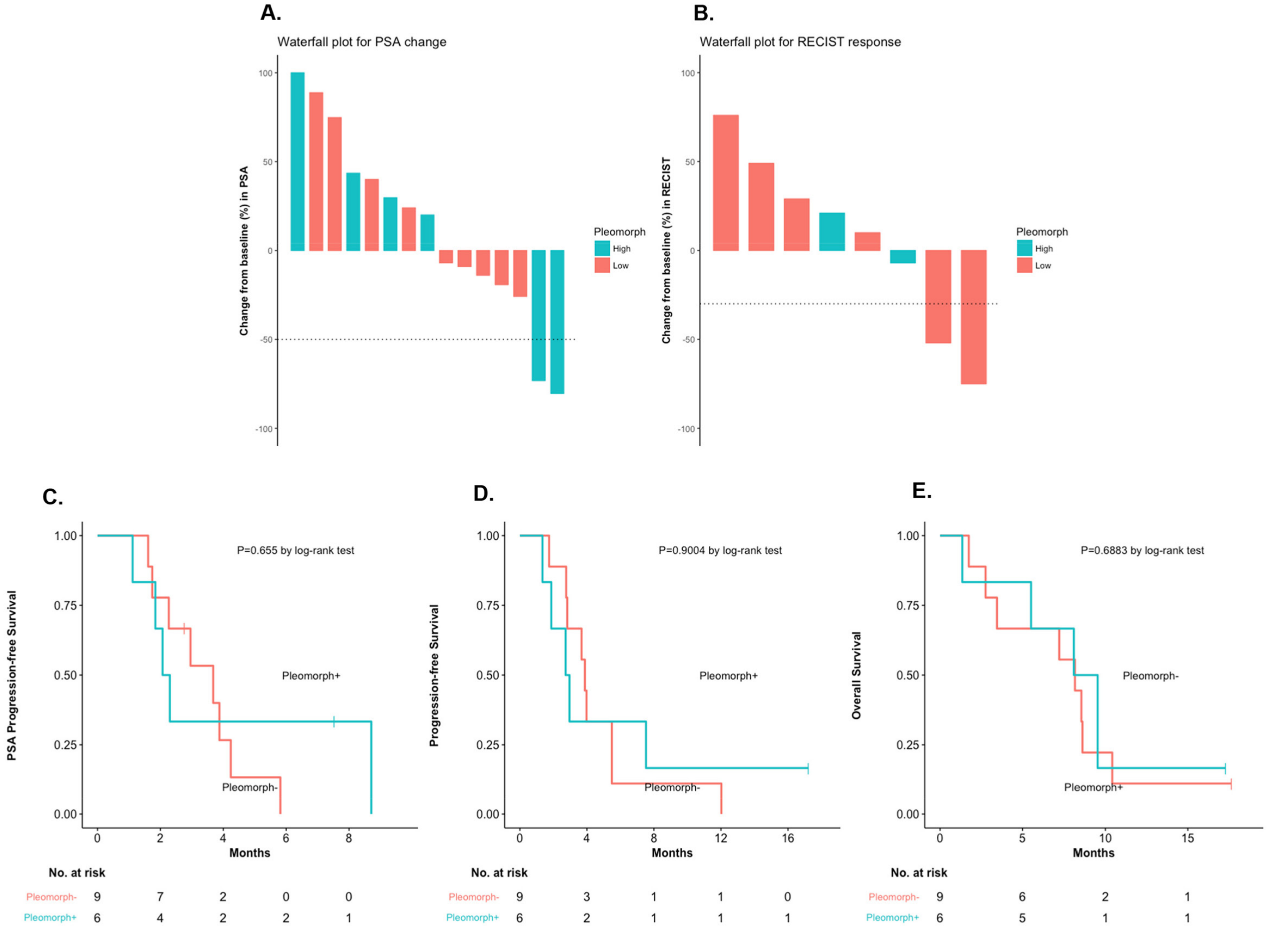

Supplementary Figure 2: Clinical outcomes, according to CTC pleomorphism. **(A)** PSA responses, according to CTC pleomorphism. **(B)** RECIST responses, according to CTC pleomorphism. **(C)** PSA-PFS, according to CTC pleomorphism [HR 0.76, 95%CI 0.22–2.58, *P*=0.66]. **(D)** PFS, according to CTC pleomorphism [HR 0.90, 95%CI 0.29–2.77, *P*=0.90]. **(E)** OS, according to CTC pleomorphism [HR 0.82, 95%CI 0.27–2.55, *P*=0.69].

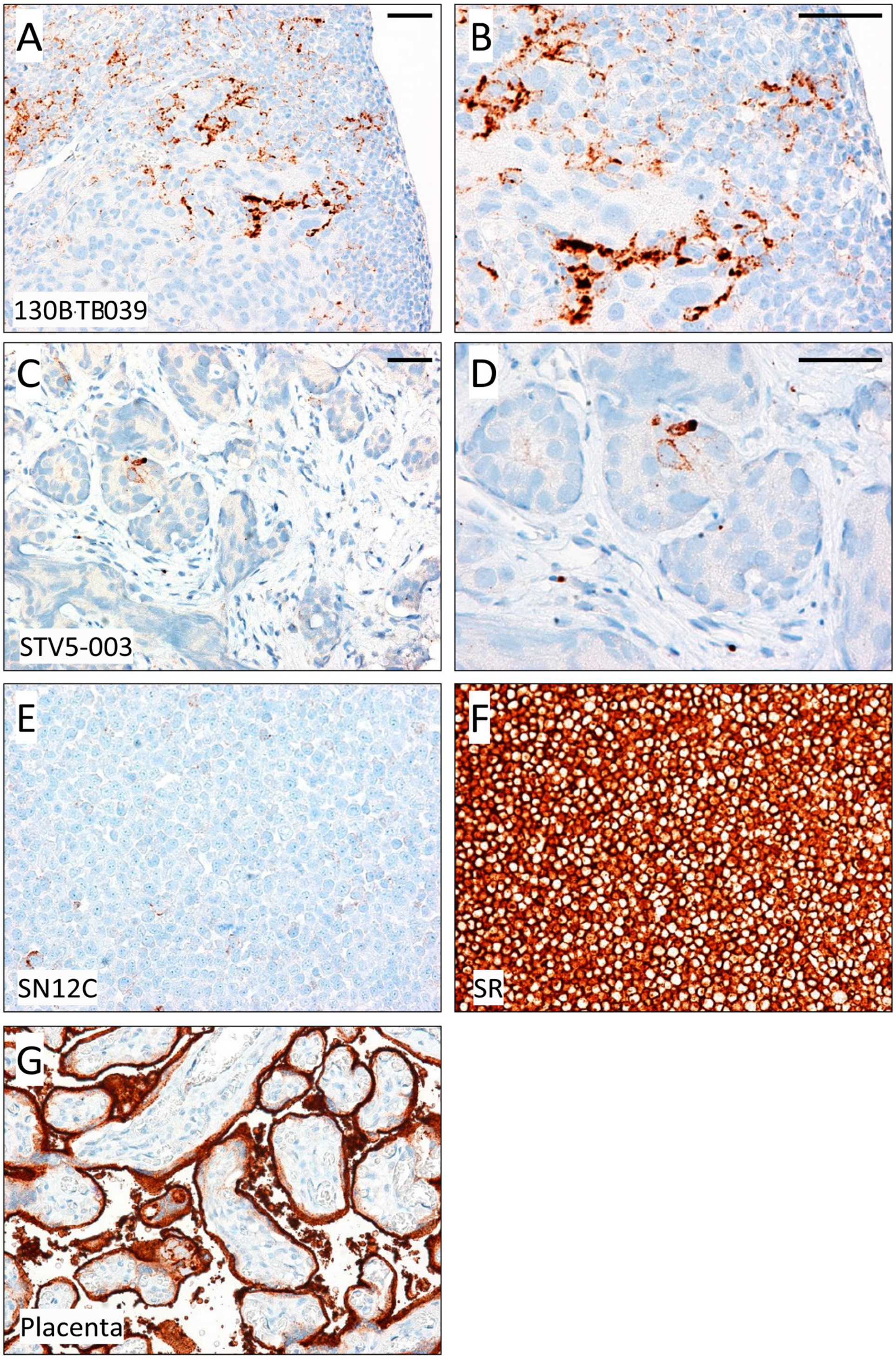

Supplementary Figure 3: Representative images of PD-L1 immunohistochemical (IHC) stains. (A) Patient #14 (*ERCC4* mutation), core-needle biopsy of lymph node, 20× magnification, and **(B)** 40× magnification. **(C)** Patient #6 (*ATM* mutation), core-needle biopsy of lymph node, 20× magnification, and **(D)** 40× magnification. **(E)** SN12C (renal cell carcinoma) cell line with deep deletion of *CD274*/*PDL1* locus; negative control. **(F)** SR (lymphoma) cell line; positive control. **(G)** Placenta; positive control.

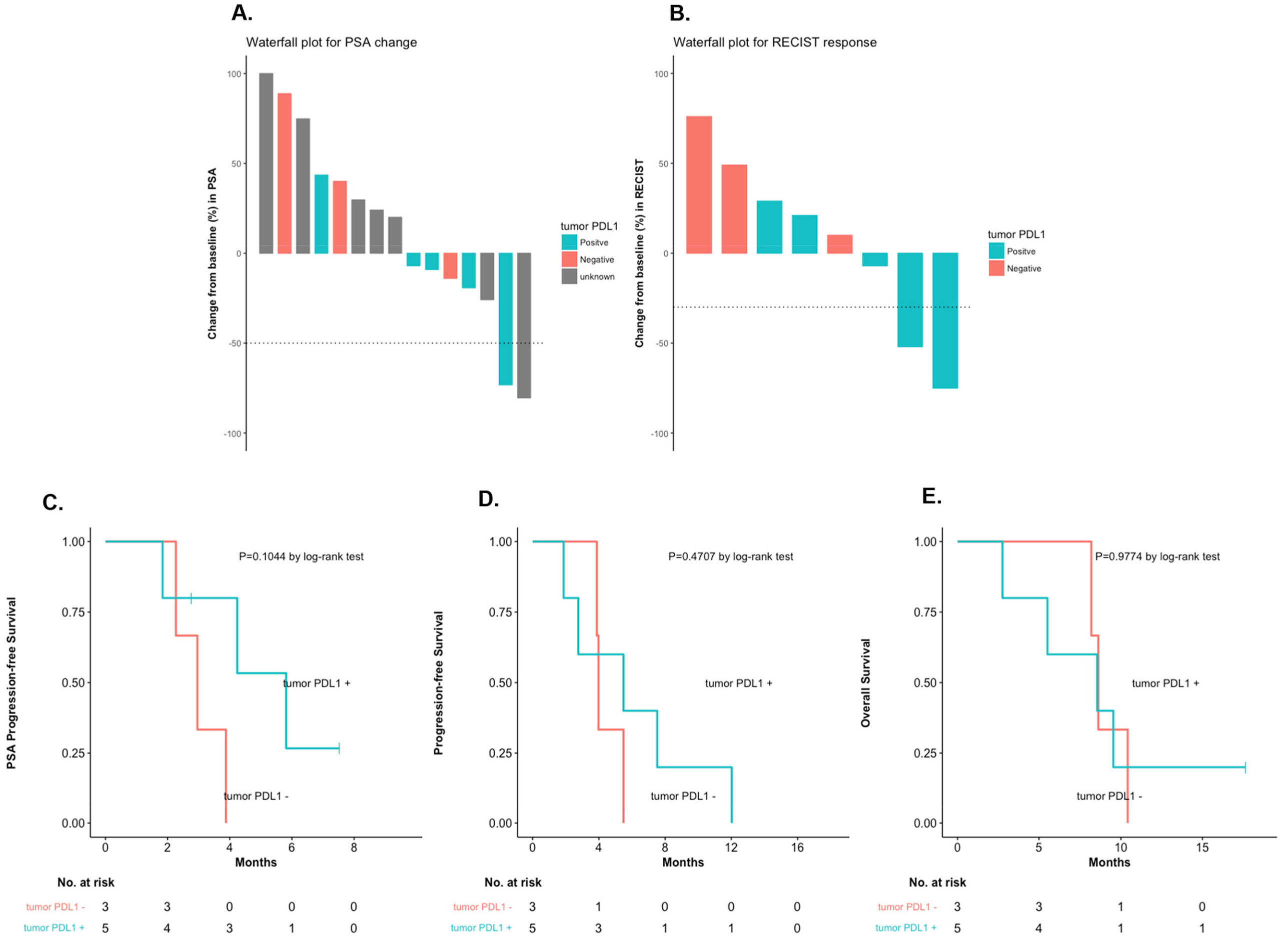

Supplementary Figure 4: Clinical outcomes, according to tumor-cell PD-L1 staining (among patients with evaluable tumor biopsies). **(A)** PSA responses, according to tumor PD-L1 staining. **(B)** RECIST responses, according to tumor PD-L1 staining. **(C)** PSA-PFS, according to tumor PD-L1 staining [HR 0.18, 95%CI 0.02–1.80, *P*=0.10]. **(D)** PFS, according to tumor PD-L1 staining [HR 0.53, 95%CI 0.10–2.75, *P*=0.47]. **(E)** OS, according to tumor PD-L1 staining [HR 1.02, 95%CI 0.23–4.62, *P*=0.98].

Supplementary Table 1: Baseline characteristics of the 15 patients treated with ipilimumab plus nivolumab, and separated by DNA-repair deficiency (DRD) status

Baseline Characteristic	All Patients (N=15)	DRD Negative (N=9)	DRD Positive (N=6)
**Age (years)**			
median (range)	65 (52-76)	65 (52-76)	65 (62-75)
**Race, N (%)**			
white	13 (86.7%)	8 (88.9%)	5 (83.3%)
non-white	2 (13.3%)	1 (11.1%)	1 (16.7%)
**ECOG performance-status score, N (%)**			
0	8 (53.3%)	4 (44.4%)	4 (66.7%)
1	7 (46.7%)	5 (55.6%)	2 (33.3%)
**Gleason sum at diagnosis, N (%)**			
≤7	4 (26.7%)	1 (11.1%)	3 (50.0%)
≥8	11 (73.3%)	8 (88.9%)	3 (50.0%)
**Baseline PSA (ng/mL)**			
median (range)	115 (31-7576)	280 (31-7576)	105 (64-6747)
**Baseline alkaline phosphatase (U/L)**			
median (range)	201 (88-967)	270 (103-967)	118 (88-687)
**Number of prior regimens for CRPC, N (%)**			
2	4 (27%)	2 (22%)	2 (33%)
3	2 (13%)	2 (22%)	0 (0%)
≥4	9 (60%)	5 (56%)	4 (67%)
**Prior treatments, N (%)**			
Abiraterone	12 (80%)	7 (78%)	5 (83%)
Enzalutamide	12 (80%)	6 (67%)	5 (83%)
Abiraterone + Enzalutamide	9 (60%)	4 (44%)	5 (83%)
Docetaxel	13 (85%)	8 (89%)	5 (83%)
Cabazitaxel	5 (33%)	3 (33%)	2 (33%)
Sipuleucel-T	7 (47%)	5 (56%)	2 (33%)
Radium-223	4 (27%)	2 (22%)	2 (33%)
**Most recent prior systemic therapy, N (%)**			
Abiraterone	3 (20%)	2 (22%)	1 (17%)
Enzalutamide	3 (20%)	1 (11%)	2 (33%)
Docetaxel	3 (20%)	2 (22%)	1 (17%)
Cabazitaxel	5 (33%)	3 (33%)	2 (33%)
Radium-223	1 (7%)	1 (11%)	0 (0%)
**Presence of bone metastases, N (%)**			
yes	15 (100%)	9 (100%)	6 (100%)
no	0 (0%)	0 (0%)	0 (0%)
**Presence of visceral metastases, N (%)**			
yes	10 (66.7%)	3 (33.3%)	2 (33.3%)
no	5 (33.3%)	6 (66.7%)	4 (66.7%)
**Presence of nodal metastases, N (%)**			
yes	7 (47.0%)	4 (44.4%)	3 (50.0%)
no	8 (74.2%)	5 (55.6%)	3 (50.0%)

Supplementary Table 2: Clinical outcomes, according to DRD mutation status

Patient no.	DRD status	DNA-repair gene mutation(s)	PSA_{50} response?	Objective (RECIST) response?	Durable PFS?	Other benefit?	Alive?
1	–	-	No	Non-measurable	No	-	No
2	–	-	No	Non-measurable	No	-	No
3	+	germline *BRCA2* (E1646Qfs*23)	No	No	No	-	No
4	+	somatic *BRCA2* (P3189H) + LOH somatic *MSH6* (E192X)	**Yes**	Mixed response	**Yes**	-	No
5	–	-	No	Non-measurable	No	-	No
6	+	somatic *ATM* (D2708N)	No	**Yes**	**Yes**	-	**Yes**
7	–	-	No	No	No	-	No
8	+	germline *BRCA2* (D3095E) + LOH somatic *FANCM* (R579H)	**Yes**	Non-measurable	**Yes**	Bone pain resolution	**Yes**
9	+	somatic *ATM* (E2039X)	No	No	No	-	No
10	–	-	No	Non-measurable	No	-	No
11	–	-	No	Non-measurable	No	-	No
12	–	-	No	Non-measurable	No	-	No
13	–	-	No	No	No	-	No
14	+	somatic *ERCC4* (D762V)	No	**Yes**	No	-	No
15	–	-	No	No	No	-	No

Supplementary Table 3: Clinical outcomes, according to CTC Shannon index (low vs. high)

	Shannon-Low (N=10)	Shannon-High (N=5)	HR (95%CI)	*P* value
**PSA_{50}, N (%) (95% CI)**	1/10 (10%) (1.8–40.4)	1/5 (20%) (3.6–62.5)	–	1.00
**ORR, N (%) (95% CI)**	0/6 (0%) (0–39.0)	2/2 (100%) (34.2–100)	–	0.036
**Durable PFS (95% CI)**	1/10 (10%) (1.8–40.4)	2/5 (40%) (11.8–76.9)	–	0.24
**PSA-PFS (mo), (95% CI)**	2.63 (1.84–NR)	4.24 (2.07–NR)	0.67 (0.23–1.99)	0.44
**PFS (mo), (95% CI)**	3.32 (1.87–NR)	5.49 (2.83–NR)	0.43 (0.15–1.22)	0.11
**OS (mo), (95% CI)**	7.67 (2.76–NR)	9.53 (8.54–NR)	0.34 (0.11–0.99)	0.069

NR: upper 95% confidence limit of survival probability not reached.

Supplementary Table 4: Clinical outcomes, according to CTC pleomorphism (low vs. high)

	Pleomorphism-Low (N=9)	Pleomorphism-High (N=6)	HR (95%CI)	*P* value
**PSA_{50}, N (%) (95% CI)**	0/9 (0%) (0–29.9)	2/6 (33.3%) (9.7–70.0)	–	0.14
**ORR, N (%) (95% CI)**	0/2 (0%) (0–65.8)	2/6 (33.3%) (9.7–70.0)	–	1.00
**Durable PFS (95% CI)**	1/9 (11.1%) (2.0–43.5)	2/6 (33.3%) (9.7–70.0)	–	0.53
**PSA-PFS (mo), (95% CI)**	3.68 (2.27–NR)	2.19 (1.84–NR)	0.76 (0.22–2.58)	0.66
**PFS (mo), (95% CI)**	3.88(2.83–NR)	2.84 (1.87–NR)	0.90 (0.29–2.77)	0.90
**OS (mo), (95% CI)**	8.18 (3.45–NR)	8.82 (5.52–NR)	0.82 (0.27–2.55)	0.69

NR: upper 95% confidence limit of survival probability not reached.

Supplementary Table 5: Clinical outcomes, according to tumor-cell PD-L1 staining (among patients with evaluable tumor biopsies)

	PD-L1 Negative (N=3)	PD-L1 Positive (N=5)	HR (95%CI)	*P* value
**PSA_{50}, N (%) (95% CI)**	0/3 (0%) (0–56.2)	1/5 (20%) (3.6–62.5)	–	1.00
**ORR, N (%) (95% CI)**	0/3 (0%) (0–56.2)	2/5 (40%) (11.8–76.9)	–	0.46
**Durable PFS (95% CI)**	0/3 (0%) (0–56.2)	2/5 (40%) (11.8–76.9)	–	0.46
**PSA-PFS (mo), (95% CI)**	2.96 (2.27–NR)	5.82 (4.24–NR)	0.18 (0.02–1.80)	0.10
**PFS (mo), (95% CI)**	3.98 (3.88–NR)	5.49 (2.76–NR)	0.53 (0.10–2.75)	0.47
**OS (mo), (95% CI)**	8.61 (8.18–NR)	8.54 (5.52–NR)	1.02 (0.23–4.62)	0.98

NR: upper 95% confidence limit of survival probability not reached.

Supplementary Table 6: Summary of treatment-emergent adverse events (AEs)

Adverse Event	All-grade (total) AEs, N (%)	Grade 1-2 AEs, N (%)	Grade 3-4 AEs, N (%)
Fatigue	14 (93.3%)	12 (80%)	2 (13.3%)
AST increased	11 (73.3%)	10 (66.7%)	1 (6.7%)
Anorexia	8 (53.3%)	7 (46.7%)	1 (6.7%)
Anemia	7 (46.7%)	6 (40.0%)	1 (6.7%)
ALT Increased	6 (40.0%)	5 (33.3%)	1 (6.7%)
Amylase increased	5 (33.3%)	4 (26.7%)	1 (6.7%)
Nausea	5 (33.3%)	5 (33.3%)	0 (0%)
Edema	4 (26.7%)	3 (20.0%)	1 (6.7%)
Hypothyroidism	4 (26.7%)	4 (26.7%)	0 (0%)
Lipase increased	4 (26.7%)	2 (13.3%)	2 (13.3%)
Diarrhea/Colitis	3 (20.0%)	1 (6.7%)	2 (13.3%)
Pneumonitis	3 (20.0%)	2 (13.3%)	1 (6.7%)
Bilirubin increased	3 (20.0%)	2 (13.3%)	1 (6.7%)
Dyspnea	3 (20.0%)	2 (13.3%)	1 (6.7%)
Vomiting	3 (20.0%)	3 (20.0%)	0 (0%)
Creatinine increased	2 (13.3%)	2 (13.3%)	0 (0%)
Malaise	2 (13.3%)	2 (13.3%)	0 (0%)
Hyperthyroidism	2 (13.3%)	2 (13.3%)	0 (0%)
Thrombocytopenia	2 (13.3%)	2 (13.3%)	0 (0%)
Weight loss	2 (13.3%)	2 (13.3%)	0 (0%)
Alk. phosphatase increased	1 (6.7%)	1 (6.7%)	0 (0%)
Neutropenia	1 (6.7%)	1 (6.7%)	0 (0%)
Constipation	1 (6.7%)	1 (6.7%)	0 (0%)
Dry eye	1 (6.7%)	1 (6.7%)	0 (0%)
Dry mouth	1 (6.7%)	1 (6.7%)	0 (0%)
Dysgeusia	1 (6.7%)	1 (6.7%)	0 (0%)
Headache	1 (6.7%)	1 (6.7%)	0 (0%)
Hepatitis	1 (6.7%)	0 (0%)	1 (6.7%)
Hypernatremia	1 (6.7%)	1 (6.7%)	0 (0%)
Stomatitis	1 (6.7%)	1 (6.7%)	0 (0%)
Pain, abdominal	1 (6.7%)	1 (6.7%)	0 (0%)
Pruritus	1 (6.7%)	1 (6.7%)	0 (0%)
Urea nitrogen increased	1 (6.7%)	1 (6.7%)	0 (0%)

Supplementary Table 7: Summary of targeted next-generation DNA sequencing analyses

Supplementary Table 7A: Summary of tumor-based targeted next-generation sequencing analyses

Patient ID	Sample ID	Pathology Based Tumor Purity (%)	Analysis Type	Gene Panel	Read Length (bp)	Bases Sequenced	Sequenced Bases Mapped to Target Regions	Average High Quality Total Coverage	Average High Quality Distinct Coverage
#13	PGDX8632T	40	Targeted NGS	CancerSELECT™ 203	100	5,866,716,400	1,881,404	1,101	843
#13	PGDX8632N	N/A	Targeted NGS	CancerSELECT™ 203	100	3,873,899,800	1,881,404	691	535
#14	PGDX6440T	50	Targeted NGS	CancerSELECT™ 203	100	7,744,979,600	1,881,404	1,061	271
#14	PGDX6440N	N/A	Targeted NGS	CancerSELECT™ 203	100	4,493,878,000	1,881,404	707	498
#3	PGDX7507T	80	Targeted NGS	CANCER*SELECT*-R™ 88	100	1,575,525,600	478,861	1,341	559
#3	PGDX7507N	N/A	Targeted NGS	CANCER*SELECT*-R™ 88	100	809,246,700	478,861	745	431
#4	PGDX7661T	80	Targeted NGS	CANCER*SELECT*-R™ 88	100	1,524,902,700	478,861	1,374	510
#4	PGDX7661N	N/A	Targeted NGS	CANCER*SELECT*-R™ 88	100	857,847,600	478,861	820	414
#6	PGDX8000T	20	Targeted NGS	CANCER*SELECT*-R™ 88	100	1,458,681,000	478,861	1,333	713
#6	PGDX8000N	N/A	Targeted NGS	CANCER*SELECT*-R™ 88	100	675,045,600	478,861	650	444
#8	PGDX8287T1	70	Targeted NGS	CancerSELECT™ 203	100	7,617,069,400	1,881,404	1,247	865
#8	PGDX8287N	N/A	Targeted NGS	CancerSELECT™ 203	100	4,363,918,600	1,881,404	387	699
#7	PGDX8291T1	70	Targeted NGS	CancerSELECT™ 125	100	2,515,684,600	979,072	712	606
#7	PGDX8291N	N/A	Targeted NGS	CancerSELECT™ 125	100	1,400,682,200	979,072	563	490
#11	PGDX8686T	70	Targeted NGS	CancerSELECT™ 203	100	6,483,047,400	1,881,404	1,177	658
#12	PGDX8687T	80	Targeted NGS	CancerSELECT™ 203	100	7,374,336,000	1,881,404	1,218	750
#12	PGDX8687N	N/A	Targeted NGS	CancerSELECT™ 203	100	4,378,785,200	1,881,404	831	608
#15	PGDX9767T1	40	Targeted NGS	CancerSELECT™ 203	100	5,728,684,800	1,881,404	1,105	345
#2	TRF127688	N/A	Targeted NGS	Foundation® Panel	N/A	N/A	N/A	N/A	N/A

Supplementary Table 7B: Summary of plasma-based targeted next-generation sequencing analyses

Patient ID	Sample ID	Analysis Type	Gene Panel	Read Length (bp)	Sequenced Bases Mapped to Target Regions	Total Genome Equivalents	Genome Equivalents per ml of plasma
#9	PGDX8188P	Targeted NGS	PlasmaSELECT™64	100	328,656	42,036	42,036
#10	PGDX8202P	Targeted NGS	PlasmaSELECT™64	100	328,656	73,454	73,454
#5	PGDX8255P	Targeted NGS	PlasmaSELECT™64	100	328,656	10,509	10,509
#1	PGDX8285P	Targeted NGS	PlasmaSELECT™64	100	328,656	37,400	37,400

Supplementary Table 7C: List of somatic sequence alterations

See Supplementary File 1
