# Supplementary material for: Ipilimumab plus nivolumab and DNA-repair defects in AR-V7-expressing metastatic prostate cancer
Source: Oncotarget. 2018 Jun 19;9(47):28561–71. doi: 10.18632/oncotarget.25564 (PMC6033362; doi:10.18632/oncotarget.25564)
Supplement: Supplementary file 2 [file oncotarget-09-28561-s002.docx]

**Supplementary Table 7C: List of somatic sequence alterations**

|  |  |  |  |  |  |  |  |
| --- | --- | --- | --- | --- | --- | --- | --- |
| **Sample ID** | **Patient ID** | **Gene** | **Transcript Accession** | **Nucleotide Position (Hg19, Genomic)** | **Amino Acid (protein)** | **Consequence** | **Mutant Reads (%)** |
| PGDX8632T | #13 | FLT3 | CCDS31953.1 | chr13:28623897-28623897_G_T | P253T | Missense | 18% |
| PGDX8632T | #13 | MED12 | CCDS43970.1 | chrX:70339657-70339657_C_A | S109Y | Missense | 31% |
| PGDX8632T | #13 | NOTCH3 | CCDS12326.1 | chr19:15289995-15289995_C_G | G1187R | Missense | 24% |
| PGDX8632T | #13 | PTEN | CCDS31238.1 | chr10:89685304-89685306_ATA_ | I67del | In-frame deletion | 21% |
| PGDX8632T | #13 | ALK | CCDS33172.1 | chr2:29445423-29445423_C_T | G1137E | Missense | 43.37% |
| PGDX8632T | #13 | AR† | CCDS14387.1 | chrX:66765955-66765955_G_A | E323K | Missense | 0.13% |
| PGDX8632T | #13 | PTEN | CCDS31238.1 | chr10:89685304-89685306_ATA_ | I67del | In-frame deletion | 30.88% |
| PGDX8188P | #9 | ATM | CCDS31669.1 | chr11:108186757-108186757_G_T | E2039X | Nonsense | 29.83% |
| PGDX8188P | #9 | DNMT3A† | CCDS33157.1 | chr2:25463218-25463218__CC | V759Gfs*21 | Frameshift | 0.22% |
| PGDX6440T | #14 | APC | CCDS4107.1 | chr5:112176152-112176152_C_A | Q1621K | Missense | N/A |
| PGDX6440T | #14 | ASXL1 | CCDS13201.1 | chr20:31022937-31022937_C_ | P808Lfs*10 | Frameshift | 16% |
| PGDX6440T | #14 | CTNNB1 | CCDS2694.1 | chr3:41266113_C_T_ | S37F | Missense | 12% |
| PGDX6440T | #14 | POLH | CCDS4902.1 | chr6:43578328-43578328_G_A | R371H | Missense | 45% |
| PGDX6440T | #14 | RUNX1 | CCDS13639.1 | chr21:36252884-36252884_C_A | D160Y | Missense | 11% |
| PGDX6440T | #14 | BRCA2 | CCDS9344.1 | chr13:32914229-32914229_T_G | C1913G | Missense | N/A |
| PGDX6440T | #14 | ERCC4 | CCDS32390.1 | chr16:14041738-14041738_A_T | D762V | Missense | N/A |
| PGDX6440T | #14 | MET | CCDS47689.1 | chr7:116403161-116403161_C_A | Q826K | Missense | N/A |
| PGDX6440T | #14 | NOTCH2 | CCDS908.1 | chr1:120483182-120483182_C_G | C1060S | Missense | N/A |
| PGDX6440T | #14 | BLM | CCDS10363.1 | chr15:91292875-91292875_C_A | P126Q | Missense | N/A |
| PGDX7507T | #3 | BRCA2 | CCDS9344.1 | chr13:32913425-32913428_AAAG_ | E1646Qfs*23 | Frameshift | 41% |
| PGDX7507T | #3 | TP53 | CCDS11118.1 | chr17:7573978-7573978_A_G | L350P | Missense | 67% |
| PGDX7507T | #3 | TP53 | CCDS11118.1 | chr17:7573991-7573991_C_A | E346X | Nonsense | 67% |
| PGDX7661T | #4 | CTNNB1 | CCDS9344.1 | chr3:41274916-41274916_C_A | S389X | Nonsense | 2% |
| PGDX7661T | #4 | DDR2 | CCDS2694.1 | chr1:162745446-162745446_G_T | D621Y | Missense | 2% |
| PGDX7661T | #4 | FANCA | CCDS32515.1 | chr16:89865635-89865635_G_T | L278I | Missense | 5% |
| PGDX7661T | #4 | KIT | CCDS3496.1 | chr4:55592076-55592076_C_A | P467Q | Missense | 2% |
| PGDX7661T | #4 | MSH6 | CCDS1836.1 | chr2:48023149-48023149_G_T | E192X | Nonsense | 3% |
| PGDX7661T | #4 | TP53 | CCDS11118.1 | chr17:7577126-7577126_T_A | E271V | Missense | 86% |
| PGDX7661T | #4 | TSC1 | CCDS6956.1 | chr9:135772929-135772929_C_A | Q898H | Missense | 2% |
| PGDX7661T | #4 | BRCA2 | CCDS9344.1 | chr13:32971099-32971099_C_A | P3189H | Missense | N/A |
| PGDX8000T | #6 | ATM | CCDS31669.1 | chr11:108205807-108205807_G_A | D2708N | Missense | 2% |
| PGDX8000T | #6 | CTNNB1 | CCDS2694.1 | chr3:41266113-41266113_C_T | S37F | Missense | 13% |
| PGDX8000T | #6 | FLT3 | CCDS31953.1 | chr13:28622514-28622514_C_A | C368F | Missense | 2% |
| PGDX8000T | #6 | TP53 | CCDS11118.1 | chr17:7579408-7579460_CCTGCTGGTG_ | A76Vfs*55 | Frameshift | 40% |
| PGDX8287T1 | #8 | FANCM | CCDS32070.1 | chr14:45633716-45633716_G_A | R579H | Missense | 30% |
| PGDX8287T1 | #8 | BRCA2 | CCDS9344.1 | chr13:32968854-32968854_C_G | D3095E | Missense | N/A |
| PGDX8291T1 | #7 | AR | CCDS14387.1 | ChrX:66931463-66931463 | L702H | Missense | 47% |
| PGDX8291T1 | #7 | AR | CCDS14387.1 | ChrX:66943552-66943552 | T878A | Missense | 2% |
| PGDX8291T1 | #7 | PTEN | CCDS31238.1 | Chr10:89717672-89717672 | R233X | Nonsense | 71% |
| PGDX8686T | #11 | CDKN1B | CCDS8653.1 | chr12:12871807-12871807_C_G | S175X | Nonsense | 36% |
| PGDX8686T | #11 | CTNNB1 | CCDS2694.1 | chr3:41266137-41266137_C_A | S45Y | Missense | 43% |
| PGDX8686T | #11 | CDKN2A | CCDS56565.1 | chr9:21970943-21970943_C_G | G139R | Missense | N/A |
| PGDX8686T | #11 | TSC2 | CCDS10458.1 | chr16:2136839-2136839_C_G | D1652E | Missense | N/A |
| PGDX8687T | #12 | JAK1 | CCDS41346.1 | chr1:65325833-65325833_G_ | P430Rfs*2 | Frameshift | 56% |
| PGDX8687T | #12 | TP53 | CCDS11118.1 | chr17:7577102-7577102_C_T | G279E | Missense | 73% |
| PGDX9767T1 | #15 | PIK3CA | CCDS43171.1 | chr3:178936091-178936091_G_A | E545K | Missense | 55% |
| PGDX9767T1 | #15 | TP53 | CCDS11118.1 | chr17:7577568-7577568_C_A | C238F | Missense | 81% |
| PGDX9767T1 | #15 | XPA | CCDS6729.1 | chr9:100447201-100447201_C_G | 673+4G>C | Splice site | 20% |
| PGDX9767T1 | #15 | KIT | CCDS3496.1 | chr4:55603425-55603425_G_T | Q927H | Missense | N/A |
| PGDX9767T1 | #15 | POLD1 | CCDS12795.1 | chr19:50905479-50905479_G_A | G203R | Missense | N/A |
| PGDX8202P | #10 | PTEN | CCDS31238.1 | Chr10:89692917-89692917_T_A | M134K | Missense | 0.40% |
| PGDX8202P | #10 | TP53 | CCDS11118.1 | Chr17:7578208-7578208_T_C | H214R | Missense | 0.08% |
| PGDX8255P | #5 | AR | CCDS14387.1 | chrX:66931463-66931463_T_A | L702H | Missense | 12.54% |
| PGDX8255P | #5 | AR | CCDS14387.1 | chrX:66943543-66943543_C_T | H875Y | Missense | 1.82% |
| PGDX8255P | #5 | AR | CCDS14387.1 | chrX:66943552-66943552_A_G | T878A | Missense | 7.04% |
| PGDX8255P | #5 | AR | CCDS14387.1 | chrX:66943582-66943582_G_C | V888L | Missense | 6.28% |
| PGDX8255P | #5 | AR | CCDS14387.1 | chrX:66943591-66943591_G_C | D891H | Missense | 25.65% |
| PGDX8255P | #5 | DNMT3A | CCDS33157.1 | chr2:25457243-25457243_G_A | R882C | Missense | 0.15% |
| PGDX8255P | #5 | DNMT3A | CCDS33157.1 | chr2:25463271-25463271_G_A | A741V | Missense | 5.68% |
| PGDX8255P | #5 | TP53 | CCDS11118.1 | chr17:7578263-7578263_G_A | R196X | Nonsense | 0.20% |
| PGDX8285P | #1 | CDKN2A | CCDS56565.1 | chr9:21974792-21974792_G_A | S12L | Missense | 0.16% |
| PGDX8285P | #1 | CTNNB1 | CCDS2694.1 | chr3:41266101-41266101_C_T | S33F | Missense | 0.08% |
| TRF127688 | #2 | PTEN | N/A | N/A | H123FS*2 | Frameshift | N/A |
| TRF127688 | #2 | RNKF43 | N/A | N/A | EXON 8 TRUNCATION | ? | N/A |
| TRF127688 | #2 | AR | N/A | N/A | G457_G464DEL | In-frame deletion? | N/A |
| TRF127688 | #2 | ATM | N/A | N/A | A78T | Missense | N/A |
| TRF127688 | #2 | CCNE1 | N/A | N/A | D130A | Missense | N/A |
| TRF127688 | #2 | CDKN2B | N/A | N/A | D86N | Missense | N/A |
| TRF127688 | #2 | FAT1 | N/A | N/A | R1654H | Missense | N/A |
| TRF127688 | #2 | LRP1B | N/A | N/A | H2567R | Missense | N/A |
| TRF127688 | #2 | ROS1 | N/A | N/A | I2251N | Missense | N/A |
| TRF127688 | #2 | SMO | N/A | N/A | R726Q | Missense | N/A |
| TRF127688 | #2 | TSC2 | N/A | N/A | E748K | Missense | N/A |
|  |  |  |  |  |  |  |  |
